# Neto2-null mice have impaired GABAergic inhibition and are susceptible to seizures

**DOI:** 10.3389/fncel.2015.00368

**Published:** 2015-09-23

**Authors:** Vivek Mahadevan, Zahra Dargaei, Evgueni A. Ivakine, Anna-Maria Hartmann, David Ng, Jonah Chevrier, Jake Ormond, Hans Gerd Nothwang, Roderick R. McInnes, Melanie A. Woodin

**Affiliations:** ^1^Department of Cell and Systems Biology, University of TorontoToronto, ON, Canada; ^2^Program in Developmental and Stem Cell Biology, Hospital for Sick Children Research InstituteToronto, ON, Canada; ^3^Department of Systematic and Evolutionary Biology, Institute for Biology and Environmental Sciences, Carl von Ossietzky University OldenburgOldenburg, Germany; ^4^Departments of Neuroscience, Biochemistry and Molecular Biophysics, Columbia UniversityNew York City, NY, USA; ^5^RIKEN Brain Sciences InstituteTokyo, Japan; ^6^Neurogenetics Group, Center of Excellence Hearing4All, School of Medicine and Health Sciences, Carl von Ossietzky University OldenburgOldenburg, Germany; ^7^Departments of Human Genetics and Biochemistry, McGill University and Lady Davis Institute, Jewish General HospitalMontreal, QC, Canada

**Keywords:** Neto proteins, KCC2, chloride, GABA, seizure, inhibition, transporter, hippocampus, auxiliary subunit

## Abstract

Neto2 is a transmembrane protein that interacts with the neuron-specific K^+^-Cl^−^ cotransporter (KCC2) in the central nervous system (CNS). Efficient KCC2 transport is essential for setting the neuronal Cl^−^ gradient, which is required for fast GABAergic inhibition. Neto2 is required to maintain the normal abundance of KCC2 in neurons, and increases KCC2 function by binding to the active oligomeric form of this cotransporter. In the present study, we characterized GABAergic inhibition and KCC2-mediated neuronal chloride homeostasis in pyramidal neurons from adult hippocampal slices. Using gramicidin perforated patch clamp recordings we found that the reversal potential for GABA (E_GABA_) was significantly depolarized. We also observed that surface levels of KCC2 and phosphorylation of KCC2 serine 940 (Ser940) were reduced in Neto2^−/−^ neurons compared to wild-type controls. To examine GABAergic inhibition we recorded spontaneous inhibitory postsynaptic currents (sIPSCs) and found that Neto2^−/−^ neurons had significant reductions in both their amplitude and frequency. Based on the critical role of Neto2 in regulating GABAergic inhibition we rationalized that Neto2-null mice would be prone to seizure activity. We found that Neto2-null mice demonstrated a decrease in the latency to pentylenetetrazole (PTZ)-induced seizures and an increase in seizure severity.

## Introduction

GABAergic inhibition in the central nervous system (CNS) is primarily mediated by postsynaptic GABA_A_ receptors (GABA_A_Rs), which are Cl^−^-permeable ion channels (Kaila, 1994). Hyperpolarizing inhibition occurs when intracellular Cl^−^ ([Cl^−^]_i_) levels are low, which allows Cl^−^ influx through the GABA_A_Rs. This low level of neuronal Cl^−^ is established by the neuron-specific K^+^-Cl^−^ cotransporter (KCC2; Rivera et al., 1999; Blaesse et al., 2009). KCC2 is a secondary active transporter that derives energy from the K^+^ gradient established primarily by the Na^+^/K^+^ ATPase to extrude Cl^−^, thereby maintaining a low [Cl^−^]_i_ in mature neurons. Once established, the low [Cl^−^]_i_ generates a strong driving force for Cl^−^ influx through GABA_A_Rs, which underlies hyperpolarizing inhibition.

KCC2 is a cation-chloride cotransporter encoded by the *SLC12* gene family (Rivera et al., 1999; Hartmann and Nothwang, 2014; Kaila et al., 2014). Because KCC2 is the only cotransporter that extrudes Cl^−^ under isotonic conditions (Acton et al., 2012), it plays a crucial role in proper neurophysiological function. KCC2 is essential for survival, as KCC2 knockout mice die at birth due to respiratory failure (Hübner et al., 2001). Moreover, chronic pain, spasticity, stress, and epileptic seizures all demonstrate impaired synaptic inhibition due to decreased KCC2 function (Coull et al., 2003; Tornberg et al., 2005; Huberfeld et al., 2007; Hewitt et al., 2009; Boulenguez et al., 2010; Puskarjov et al., 2012; Gagnon et al., 2013).

KCC2 expression and function are regulated by multiple posttranslational mechanisms including phosphorylation, oligomerization, calpain-mediated cleavage and association with lipid rafts (Blaesse et al., 2006; Hartmann et al., 2009; Rinehart et al., 2009, 2011; Watanabe et al., 2009; Lee et al., 2011; Puskarjov et al., 2012; Zhou et al., 2012). Recently, we demonstrated that native-KCC2 exists in a physical and functional complex with components of excitatory neurotransmission, and proper KCC2 expression and function requires interaction with the kainate receptor subunit GluK2 (Mahadevan et al., 2014), and its auxiliary subunit Neto2 (Ivakine et al., 2013). Neto2 is a transmembrane protein named for its similarity to neuropilin and tolloid like protein (Zhang et al., 2009; Copits and Swanson, 2012; Straub and Tomita, 2012; Tang et al., 2011; Tomita and Castillo, 2012). The Neto2-KCC2 interaction is required to maintain the normal abundance of KCC2 in neurons, and increases KCC2 function by binding to the active oligomeric form of this cotransporter. Despite the important roles that Neto2 plays in proper neurophysiological function, the behavioral consequence of KCC2 dysfunction in adult Neto2-deficent mice has not been characterized.

In the present study, we tested the hypothesis that hippocampal neurons of adult Neto2-null mice would have impaired KCC2 function, and as a result be susceptible to seizure induction. We tested this hypothesis by comparing Neto2-null mice to wild-type and KCC2-deficient mice. We found that Neto2-null mice have a decreased latency to seizure induction, and an increased seizure severity compared to wild-types. In addition, we observed a decrease in surface oligomeric KCC2 levels, and serine 940 (Ser940) phosphorylation, and a depolarized E_GABA_ in the hippocampus of Neto2^−/−^ mice. Additionally we also observed significant reductions in both spontaneous inhibitory postsynaptic current (sIPSC) amplitude and frequency in CA1 neurons from Neto2^−/−^ mice. Taken together we conclude that Neto2 critically contributes to diverse aspects of GABAergic inhibition including GABAergic synaptic drive and postsynaptic KCC2 regulation, which together render Neto2-null mice susceptible to seizures.

## Materials and Methods

### Animals

All experimental procedures were performed according to the Canadian Council for Animal Care guidelines. The experimental protocols were approved by the University of Toronto Animal Care Committee. All efforts were made to minimize animal suffering, to reduce the number of animals used, and to utilize alternatives to *in vivo* techniques. Mice were maintained in either the Biological Sciences Facility in the Faculty of Arts and Science at the University of Toronto or the Toronto Center for Phenogenomics. The following animals were used: C57/Bl6 mice (Charles River; Wilmington, MA, USA); *Neto2^−/−^* mice congenic with C57 (Ivakine et al., 2013); *KCC2–1b^+/−^* on a C57/129SVe genetic background (Woo et al., 2002).

### Hippocampal Slice Preparation

Hippocampal slices (400 μM) were prepared as similarly described for rodents (Ormond and Woodin, 2009) from 1–4 month old mice, using a Vibratome 1000 plus in modified artificial cerebrospinal fluid [(aCSF, in mM: 180 sucrose, 25 sodium bicarbonate, 25 glucose, 2.5 KCl, 1.25 sodium phosphate, 2 MgCl_2_, 1 CaCl_2_, 0.4 sodium ascorbate, and 3 sodium pyruvate, and saturated with 95% O_2_/5% CO_2_ (pH 7.4, osmolarity ~305 mOsm)]. Slices recovered in 35–37°C ACSF composed of (in mM) 125 NaCl, 25 sodium bicarbonate, 25 glucose, 2.5 KCl, 1.25 sodium phosphate, 1 MgCl_2_, 2 CaCl_2_ and saturated with 95% O_2_/5% CO_2_ (pH 7.4, osmolarity ~305 mOsm) for 1 h.

### Electrophysiology

Electrophysiological recordings from hippocampal slices were made in oxygenated aCSF at 35–37°C from CA1 pyramidal cells as previously described (Ormond and Woodin, 2009). Recording pipettes were pulled from thin-walled borosilicate (TW-150 F, World Precision Industries; Sarasota, Florida) to resistances of 7–12 MΩ with a Sutter Instruments P-87 (Novato, CA, USA). Pipettes were filled with an internal solution containing 150 mM KCl, 10 mM HEPES, and 50 μg/ml gramicidin (pH 7.4, 300 mOsm). Signals were amplified using an Axon Instruments Multiclamp 700b and digitized using an Axon Instruments Digidata 1322a (Molecular Devices; Sunnyvale, CA, USA). Extracellular stimulation was applied through a whole-cell recording pipette filled with ACSF, at a baseline recording frequency of 0.03 Hz. 6-cyano-7-nitroquinoxaline-2,3-dione (CNQX) was used to block glutamatergic transmission for recordings of isolated inhibitory synaptic potentials (IPSPs). N-methyl-D-aspartate receptors (NMDARs) were not pharmacologically antagonized as we previously demonstrated no difference in E_GABA_ in the presence or absence of DL-2-amino-5-phosphonovaleric acid (AP-5; Ormond and Woodin, 2011). Recordings commenced when the access resistance dropped below 40 MΩ and stabilized (within 10%). E_GABA_ was determined in current clamp mode by evoking IPSPs while step depolarizing the membrane potential (at 30 s intervals); during each current step an IPSP was evoked in order to generate an IPSP-Vm. A linear regression of the IPSP amplitudes was then used to calculate the membrane potential dependence of IPSPs. The intercept of this line with the abscissa was taken as E_GABA_. Values have not been corrected for the liquid junction potential of −2 mV.

GABA receptor-mediated sIPSCs were measured in whole-cell configuration. Neuronal membranes were stepped to a holding potential of +10 mV and ionotropic glutamate receptor-mediated currents blocked with 10 μM CNQX and 50 μM AP-5. A separate template was created for each recording by averaging a number of hand-picked IPSCs. Data was extracted using template-based detection. sIPSCs were analyzed offline using the Clampfit 9.2 and subsequently verified manually for precision.

### Tl^+^-Flux Assay

**Figure 1:**
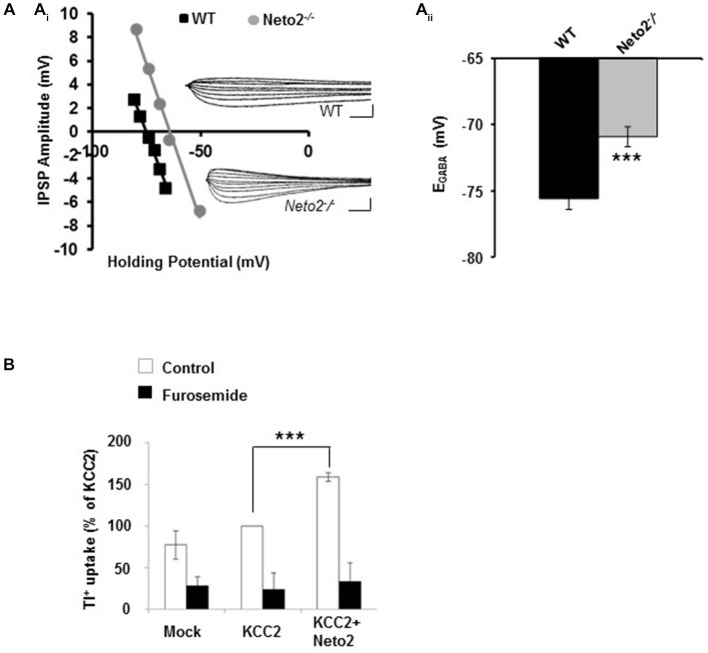
**Hippocampal pyramidal neurons from adult Neto2-null mice have depolarized E_GABA_. (A_i_)** Example of gramicidin perforated patch clamp recordings obtained from CA1 pyramidal neurons in acute hippocampal slices prepared from Neto2-null mice (*n* = 15 neurons, gray) and wild-type mice (*n* = 21 neurons, black). The inhibitory postsynaptic potential (IPSP) amplitude was plotted against the holding potential of the postsynaptic membrane, and the intercept of this curve with the *x*-axis was taken as E_GABA_. Insets: traces of IPSPs for the examples shown. Scale bars: 5 mV, 10 ms. **(A_ii_)** Summary of all experiments similar to **(A_i_). (B)** Tl^+^-uptake assay performed in HEK-293 cells (*n* = 4 sets), in the absence (white bars) and presence of 2 mM furosemide (black bars). ***p < 0.001.

Transport activity was determined by measuring Cl^−^-dependent uptake of Tl^+^ in HEK-293 cells. Uptake measurements were done as described previously (Delpire et al., 2009; Hartmann et al., 2010; Weber et al., 2014). Cells were transiently transfected with empty plasmid or pcDNA3.1 KCC2 or pcDNA3.1 KCC2 alone + pcDNA3.1 Neto2 constructs. Briefly, 150 μl of Opti-MEM (Invitrogen), 6 μl of TurboFect (Fermentas, Karlsruhe, Germany), and appropriate cDNA were mixed and incubated for 20 min at room temperature prior to transfection (the total cDNA transfected were 750 ng, and the ratio of KCC2: Neto2 transfection was in 1:1 ratio). Twenty four hours after transfection, HEK-293 cells were plated in a black-walled 96-well culture dish (Greiner Bio-One, Frickenhausen, Germany) at a concentration of 100,000 cells/well. The HEK-293 cells were processed for flux measurements by replacing the medium with 80 μl of preincubation buffer (100 mM *N*-methyl-D-glucamine, 5 mM KCl, 2 mM CaCl_2_, 0.8 mM MgSO_4_, 5 mM glucose, 5 mM HEPES, pH 7.4) with or without 2 μM FlouZin-2 AM dye (Invitrogen) plus 0.2% (w/v) Pluronic F-127 (Invitrogen). After incubation for 48 min at room temperature, cells were washed three times with 80 μl of preincubation buffer and incubated for 15 min with 80 μl of preincubation buffer plus 0.1 mM ouabain to block Na^+^/K^+^ ATPases. Thereafter, the culture dish was inserted into a fluorometer (Fluoroskan Accent, Thermo Scientific, Bremen, Germany), and the wells were injected with 40 μl of 5× thallium stimulation buffer (12 mM Tl_2_SO_4_, 100 mM *N*-methyl-D-glucamine, 5 mM HEPES, 2 mM CaSO_4_, 0.8 mM MgSO_4_, 5 mM glucose, pH 7.4). The fluorescence across the entire cell population in a single well was measured in a kinetic dependent manner (excitation 485 nm, emission 538 nm, 1 frame in 4 s in a 200-s time span). The activity was calculated with the initial values of the slope of Tl^+^-stimulated fluorescence increase by using linear regression. At least two independent DNA preparations were used per construct, giving similar results. At the end of each experiment, Tl^+^ flux was blocked in the presence of 2 mM furosemide, to demonstrate the specificity of the KCC2 transporter activity. Experiments in Figure 1B are representative results from four independent biological replicates.

### Induction and Scoring of PTZ-Induced Seizures

Induction of seizures by pentylenetetrazole (PTZ) was performed similarly as previously described (Tornberg et al., 2005). PTZ (Sigma-Aldrich; St. Louis, MO, USA) was administered intraperitoneally at 50 mg/kg in 1 × PBS to 9–13 week old female mice. Each mouse was observed in isolation for 10 min following PTZ injection. Seizures were scored as previously described (Löscher and Nolting, 1991). Seizure latency was recorded as the time from PTZ injection to seizure occurrence (loss of righting reflex). Seizure severity was scored as mild, moderate, or severe according to the following criteria: mild seizures were defined as a loss of righting reflex lasting less than 5 s; moderate seizures were scored if the loss of righting reflex was accompanied with rapid repetitive clonic (or clonic-tonic) convulsions of forelimbs and hindlimbs lasting less than 10 s; severe seizures were scored if convulsions lasted longer than 10 s or when a moderate seizure was followed by a second moderate seizure within 1 min of the onset of the first seizure.

### Surface Biotinylation Assay

**Figure 2:**
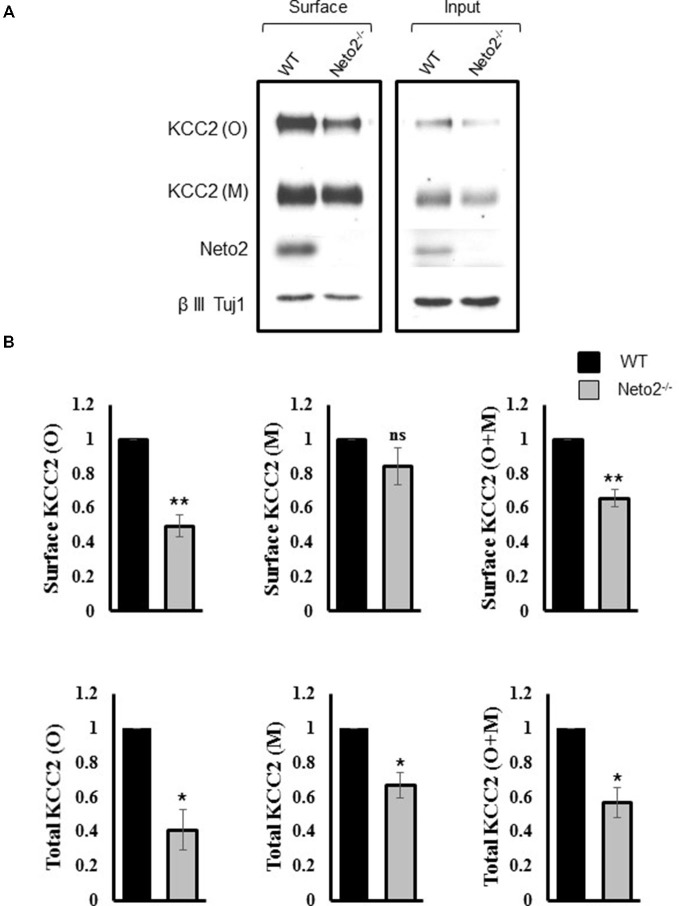
**Hippocampal neurons from Neto2-null mice have decreased surface KCC2 levels. (A)** Representative immunoblots of KCC2 levels from the surface and total fractions, from wild-type and Neto2-null neurons. The first two lanes in **(A)** correspond to biotinylated surface proteins (50 μg) recovered from the neutravidin beads. The last two lanes correspond to total proteins (5 μg). **(B)** Summary figures showing levels of surface and total KCC2 levels, normalized to β III Tuj1 in Neto2 null neurons, relative to that of wild type (*n* = 3). Summary figures represent mean ± sem. *p < 0.05, **p < 0.01.

Biotinylation studies were performed as previously described (Mahadevan et al., 2014) with modifications. Briefly, DIC10–11 hippocampal neurons from wild type and Neto2-null mice were washed three times with ice cold 1 × PBS then incubated for 30 min on ice in 1 × PBS containing 1 mg/ml biotin (Pierce Protein Research Products, Thermo Scientific). Cultures were then incubated in 10 mM Tris/HCl [pH 7.4], washed three times with 1 × PBS, homogenized with 0.5 mL of RIPA buffer [50 mM Tris·HCl, pH 7.4, 150 mM NaCl, 1 mM EDTA, 1% Nonidet P-40, 0.1% SDS, 0.5% DOC, protease inhibitors and phosphatase inhibitor mixture (Roche)], and incubated on ice for 30 min. The homogenate was centrifuged, supernatant was collected and quantified using the BioRad DC protein quantification kit. 50 μg of total protein in a total volume of 400 μL was mixed with 100 μL of a 50% slurry of Neutravidin beads (Pierce Protein Research Products, Thermo Scientific) and rotated for 1 h at 4C. The beads (first bound fraction) were harvested by centrifugation and washed three times in RIPA buffer. After the last wash all buffer was thoroughly removed from beads, and the biotin-bound and total input fractions were denatured in 6 × SDS sample buffer containing DTT at 37°C for 1 h before resolving them on onto 6% SDS-PAGE. Subsequent immunoblot analysis was performed as described elsewhere (Mahadevan et al., 2014). Experiments in Figure 2 are representative results from three independent biological replicates.

### 2D-BN PAGE

All biochemical preparations, centrifugations and 1D-BN-PAGE were performed at 4°C. Native-membrane fractions were prepared similarly as described (Swamy et al., 2006; Mahadevan et al., 2014). Briefly, mouse hippocampus (~P30) were homogenized on ice in PBS using a glass-Teflon homogenizer, followed by a brief low-speed centrifugation. Soft pellets were resuspended in ice-cold lysis buffer [Bis-tris, 20 mM, pH7; EDTA, 0.2 mM; sucrose, 300 mM; protease and phosphatase inhibitor mixture (Roche)], homogenized, and centrifuged for 30 min at 25,000 × g. The membrane pellets were resuspended in solubilization buffer (4× w/v) [Bis-tris, 20 mM, pH7; 6-aminocaproic acid, 500 mM; NaCl, 20 mM; EDTA, 0.2 mM; glycerol, 10%; iodoacetamide, 25 mM; 1% dodecyl-maltoside or 1% digitonin or 1.5% C_12_E_9_; protease and phosphatase inhibitor mixture], solubilized for 3 h on a rotating platform at 4°C, and centrifuged for 1 h at 25,000 × g. Ten to twenty μg of proteins and native-equine ferritin were mixed with BN-PAGE sample buffer (bis-Tris, 200 mM; 6-aminocaproic acid, 1 M; Coomassie blue G250, 5%; sucrose, 30%; iodoacetamide, 25 mM) and loaded on searate lanes on a linear 5% home-made Bis-Tris gels. Samples were separated in a 1D-BN-PAGE as described (Swamy et al., 2006; Mahadevan et al., 2014), using separate cathode buffer (bis-Tris, 15 mM, pH7; Tricine, 50 mM; Coomassie blue G250, 0.02%), and anode buffer (Bis-Tris 50 mM, pH7). After the 1D gel run completion, the gels were imaged for subsequent calculation of approximate molecular mass prediction.

**Figure 3:**
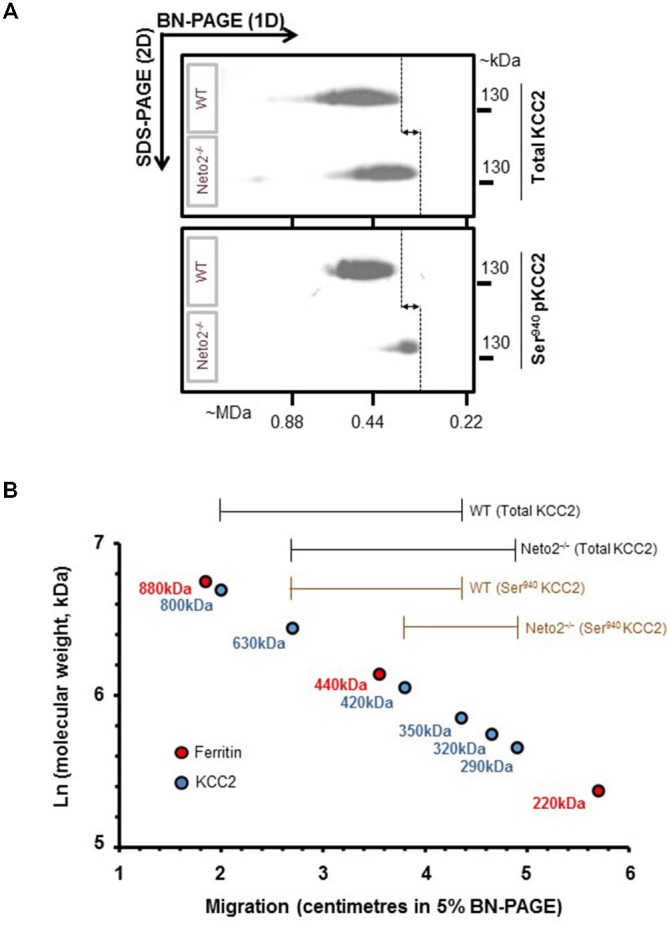
**Native-KCC2 from adult Neto2-null neurons exhibit decreased phosphorylated Ser940 status. (A)** Two-dimensional BN-PAGE separation of native, total KCC2 and Ser940-phosphorylated KCC2 complexes from ~P30 hippocampus solubilized with C_12_E_9_, from WT or Neto2^−/−^ mice; gel separations were immunoblotted with the antibodies indicated on right (total KCC2, phosphorylated Ser940KCC2). This blot is a representative example of three independent biological replicates. Majority of total KCC2 is phosphorylated at Ser940 residue in WT lysates while only a small proportion of total KCC2 is phosphorylated at Ser940 residue in Neto2-null lysates. The dashed lines and arrows indicate the decrease in molecular weight of native-KCC2 in Neto2-null lysates compared to WT. **(B)** Calibration of the 2D-BN-PAGE separation using native-ferritin marker composed of distinct molecular mass (220, 440 and 880 kDa). This allowed for determination of the approximate molecular mass of the native-KCC2 assemblies, during the absence or presence of Neto2. The existing Neto2-independent native Ser940 KCC2 migrates at a lower molecular weight in Neto2-null lysates when compared to WT lysates. This indicates that Neto2 is an essential component of Ser940 phosphorylated native-KCC2.

2D BN-PAGE analysis were performed as described (Schwenk et al., 2012; Mahadevan et al., 2014) with minor modifications. After the completion of the gel run, excised BN-PAGE lanes were equilibrated in Laemmli buffer containing SDS and DTT for 30 min at room temperature to denature the native proteins. After a brief rinse in SDS-PAGE running buffer, the excised BN-PAGE lanes were placed on a 6% SDS-PAGE gel for separation in the second dimension. After standard electroblotting of SDS-PAGE-resolved samples on PVDF membrane, the blots were subjected to western blotting analysis with Ms anti-KCC2 (developmental studies hybridoma bank, clone N1/12), Rb anti-Ser940 phosphorylated KCC2 (Phosphosolutions, p1551–940). The Ln (native-ferritin molecular weights 220 kDa, 440 kDa, 880 kDa) were plotted against the distance of migration of native-ferritin marker (in cms) in a 5% 1D BN-PAGE. Using this as a standard, the approximate molecular weights of Ser940 phosphorylated native-KCC2 were deduced after immune-blotting. Experiments in Figure 3 are representative results from three independent biological replicates.

### Statistical Analysis

**Figure 4:**
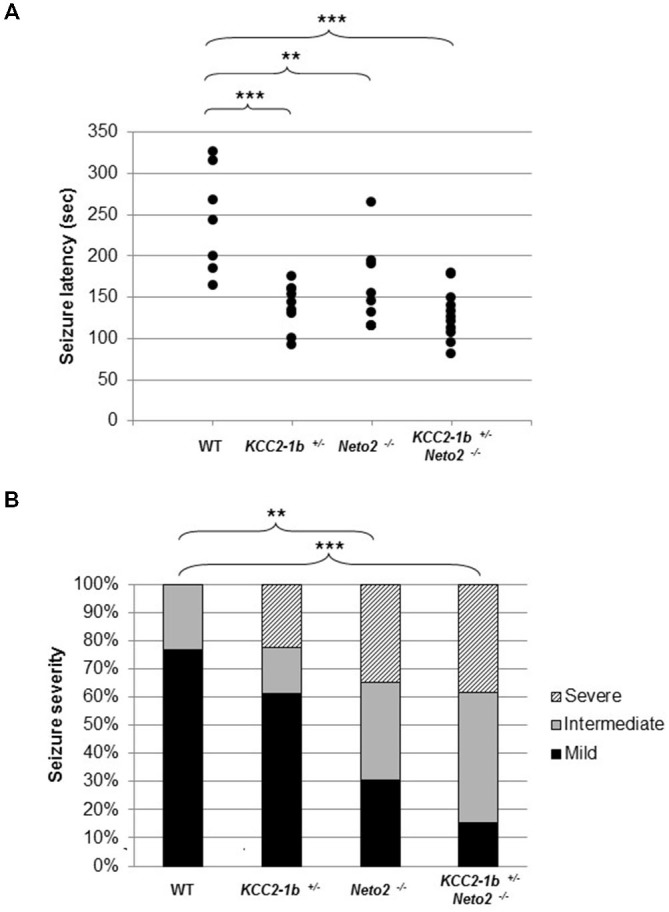
**Neto2-null mice have a decreased latency to PTZ-induced seizures and increased PTZ-induced seizure severity. (A)** Time of onset of the first seizure (seizure latency; measured in seconds) was plotted for individual mouse strains wild type (*n* = 7), *Neto2*^−/−^ (*n* = 9), and *Kcc2–1b*^+/−^ (*n* = 9). Similar results were obtained in two independent experiments. **p < 0.01, ***p < 0.001. **(B)** The severity of the PTZ-induced seizures was determined for each mouse strain. Every mouse was assigned a seizure severity score (mild, intermediate, or severe). For every strain, the percentage of mice with a given score is indicated. Differences between strains were analyzed using Cochran-Armitage Trend Test **z < 0.01, ***z < 0.001.

Results are given as mean ± SEM. Statistical significance for Figures 1A_ii_,B, 2B, 5B were tested using the Student’s *t* test; statistical significance was determined as follows: *p < 0.05, **p < 0.01, ***p < 0.001. For Figure 4, differences between strains was analyzed using the Cochran-Armitage Trend Test (**z < 0.01).

**Figure 5:**
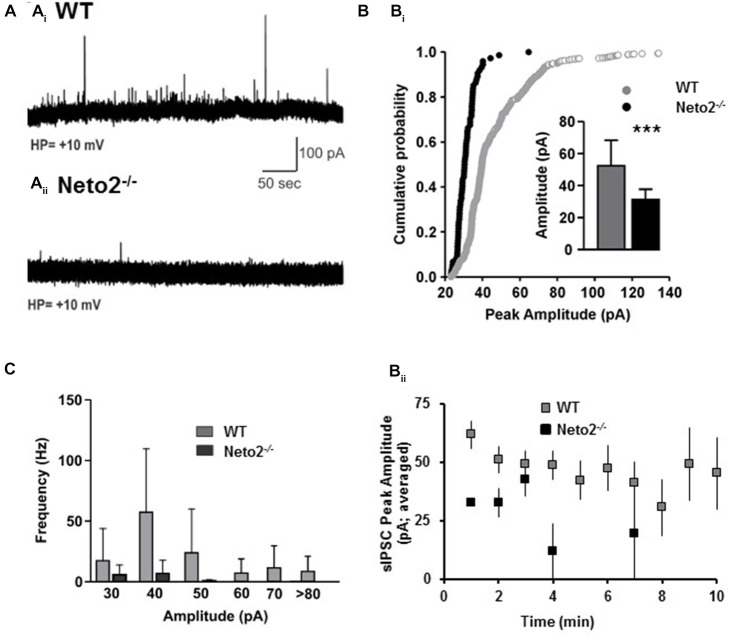
**Neto2-null CA1 neurons have reduced spontaneous IPSCs. (A)** Example of sIPSC traces obtained from CA1 pyramidal neurons in acute hippocampal slices prepared from wild-type mice **(A_i_)**, or Neto2-null mice **(A_ii_)**; WT: *n* = 7 neurons; *Neto2*^−/−^: *n* = 8 neurons; across three animals. **(B_i_)** Cumulative probability plot indicating the distribution of sIPSC peak amplitude; Inset: Histograms (mean ± S.E.) showing the sIPSC peak amplitude from the two genotypes. ***p < 0.001. **(B_ii_)** Average sIPSC peak amplitude plotted against time course analyzed. **(C)** Histograms (mean ± S.E.) showing the sIPSC frequency of the events of different amplitudes.

## Results

### KCC2-Mediated Cl^−^-Homeostasis is Impaired in Neurons from Neto2^−/−^ Adult Hippocampus

We previously demonstrated that Neto2-null mice have reduced KCC2-mediated Cl^−^-extrusion (Ivakine et al., 2013). Because efficient Cl^−^-extrusion from neurons is required for hyperpolarizing GABAergic inhibition, we rationalized that hippocampal neurons from adult Neto2-null mice would have reduced inhibition that increased their susceptibility to seizures. To test this hypothesis we first recorded from putative pyramidal neurons in hippocampal slices obtained from 1–4 month old adult mice. We assessed synaptic inhibition by recording the reversal potential for GABA (E_GABA_), which provides a measure of both Cl^−^-extrusion and the strength of GABAergic inhibition. We recorded E_GABA_ using the gramicidin (25 μg/ml) perforated patch clamp technique; gramicidin forms pores which are impermeable to Cl^−^, permitting reliable recordings of GABAergic currents (Kyrozis and Reichling, 1995; Woodin et al., 2003). We stimulated putative interneurons in the *stratum radiatum* to release GABA and recorded from putative pyramidal neurons in the *stratum pyramidale* in current clamp mode, as we have done previously to measure changes in E_GABA_ (Ormond and Woodin, 2009, 2011; Takkala and Woodin, 2013). These recordings were made in the presence of CNQX (10 μM) to block AMPA/KA receptor-mediated transmission. We found that *Neto2*^−/−^ neurons had significantly depolarized E_GABA_, in comparison to wild type neurons (Figure 1A; wild type: −75.58 ± 0.81 mV, *n* = 21; *Neto2^−/−^*: −70.91 ± 0.76 mV, *n* = 15; *p* < 0.001). These recordings suggest that in the absence of Neto2, KCC2 function is impaired in hippocampal pyramidal neurons from adult mice. To examine whether Neto2 is directly sufficient to regulate KCC2 function we performed a Tl^+^-flux assay in HEK-293 cells. We transfected cells with plasmids encoding either KCC2 or KCC2+Neto2. We observed that in the presence of Neto2, KCC2-mediated Tl^+^ flux significantly increased when compared to KCC2 alone (Figure 1B; *n* = 4 independent experimental replications, *p* = 0.0004). Thus, in addition to regulating native KCC2 function, Neto2 can also regulate recombinant KCC2-mediated ion transport.

### Neto2-Null Neurons have a Decrease in Surface Levels of KCC2

We previously reported an overall decrease in total KCC2 abundance in Neto2^−/−^ hippocampal neurons (Ivakine et al., 2013). However, KCC2-dependent Cl^−^ extrusion in neurons is most dependent on KCC2 expression in the neuronal membrane (i.e., the surface pool). Therefore, we hypothesized that Neto2-lacking neurons would have a decrease in surface KCC2 levels, in addition to the decrease in total levels previously reported (Ivakine et al., 2013). We performed a biotinylation assay in order to quantify the surface pool of KCC2. Consistent with our previous observations, we saw a significant reduction in both monomeric and oligomeric KCC2 in the total inputs (*p* < 0.05, *n* = 3; Figure 2A, right panel, Figure 2B), and a significant decrease in oligomeric KCC2 levels in the neuronal surface pool (*p* < 0.01, *n* = 3; Figure 2A, left panel, Figure 2B). Interestingly the decrease in KCC2 surface levels was not greater than the decrease in total KCC2 levels (quantification not shown), suggesting that Neto2 might be required for KCC2 biogenesis or total protein stability.

### Neto2-Null Mice have a Decrease in Native-KCC2 Pool of Phosphorylated Ser940

KCC2 function is critically dependant on phosphorylation of Ser940 residue (Lee et al., 2007; Kahle et al., 2013). To test whether Ser940 phosphorylation of native-KCC2 is compromised in Neto2-null neurons, we examined the oligomeric KCC2 pool using a well-validated phosphorylated Ser940 KCC2 antibody (Lee et al., 2011) using a BN-PAGE assay. BN-PAGE allows the separation of native membrane proteins and the phosphorylation status of native KCC2 oligomers. First, in line with our previous study (Mahadevan et al., 2014), mature neuronal total KCC2 from WT lysates migrate predominantly between ~350–800 kDa, but there is a modest decrease in the molecular mass of total KCC2 from Neto2-null lysates, which migrate between ~290–630 kDa (Figure 3A, top panel). Since Neto2 is a critical component of oligomeric KCC2 (Ivakine et al., 2013), the drop in molecular weight of native-KCC2 in Neto2-null lysates indicate the loss of Neto2 within the complex. Next, we assessed the oligomeric state of native Ser940 phosphorylated KCC2. While we found that the majority of total KCC2 was phosphorylated at Ser940 in WT hippocampal neurons, Neto2-null neurons had a dramatic reduction in the abundance of phosphorylated Ser940 KCC2 levels (Figure 3A, bottom panel). In addition, we observed that while the phosphorylated Ser940 KCC2 in WT lysates migrated between ~350–630 kDa, the phosphorylated Ser940 KCC2 in Neto2-null lysates migrated at a much lower molecular weight between ~290–420 kDa (Figure 3B). This strongly suggests that in addition to being an essential component of the total-KCC2 pool, Neto2 also serves to maintain the abundance of phosphorylated KCC2 at Ser940 residue *in vivo*.

### Neto2-Null Mice have a Decrease Latency to PTZ-Induced Seizures

Impaired KCC2 function and even modest depolarization of resting E_GABA_ disrupt the network excitation: inhibition balance, which contributes to the generation of seizures (Woo et al., 2002; Tornberg et al., 2005; Huberfeld et al., 2007; Kahle et al., 2008, 2014; Puskarjov et al., 2014; Silayeva et al., 2015). In particular, aberrant phosphorylation of Ser940-KCC2 was recently demonstrated to contribute towards the severity of status epilepticus (Silayeva et al., 2015). Thus, we hypothesized that the depolarization of E_GABA_ we observed in hippocampal pyramidal neurons, in combination with reduced KCC2 Ser940 phosphorylation in Neto2-null adult mice would increase the susceptibility and/or the severity of chemically-induced seizures.

We tested this hypothesis by inducing seizures with PTZ. We selected PTZ because it has been previously reported that mice with reduced levels of KCC2 (*Kcc2–1b^+^*) are more susceptible to PTZ-induced seizures (Woo et al., 2002; Cremer et al., 2009). We first verified that an injection of 50 mg/kg of PTZ intraperitoneally induced a seizure in wild type mice; we found that seizures were induced after 243.4 ± 63.4 s following injection (Figure 4; *n* = 7). We then confirmed previous reports that *Kcc2–1b^+/−^* mice have an increased susceptibility to PTZ-induced seizures, and found that there was a significant decrease in the latency to seizure compared to wild type mice (Figure 4; latency to seizure 138.7 ± 27.9 s; *n* = 9; *p* < 0.001 compared to WT). Because Neto2 null mice have reduced Ser940 phosphorylated native-KCC2 we rationalized that they would also have a decrease in the latency to seizures compared to wild type; we found this rationalization was correct (Figure 4; latency to seizure 158.7 ± 50.2 s; *n* = 9; *p* < 0.01 compared to WT). If the decrease in seizure latency in Neto2 null mice was due to a KCC2 deficiency, we would expect that in neurons harboring only one copy of *Kcc2–1b*, the existing KCC2 function would be further compromised if the neurons also lack *Neto2*. We tested this hypothesis by breeding *Kcc2–1b^+/−^* Neto2^−/−^ mice and assessed seizure latency in these mice. While the latency was significantly different from WT, it did not differ significantly from either *Kcc2–1b^+/−^* or *Neto2*^−/−^ mice.

### Neto2-Null Mice have Increased PTZ-Induced Seizure Severity

We next asked whether in addition an increase in latency to seizure, the loss of Neto2 also produced alterations in seizure severity. We assessed seizure severity by classifying the seizures as mild, intermediate and severe. In this assay, the majority of the wild type mice exhibited mild seizures (10/13), whereas a large proportion of Neto2-null and double mutant mice displayed seizures classified as intermediate and severe (16/23 and 22/26, respectively; Figure 4B). We used the Cochran-Armitage Trend Test to compare the seizure severity trend between strains of mice, and found that both Neto2-null and double mutant mice were statistically different from the wild type strain (*z* = 0.0032 and *z* = 0.0002, respectively). KCC2 heterozygous mice showed a trend toward a higher seizure severity compared to the wild type strain, but it did not reach statistical significance (*z* = 0.14).

### Neto2-Null Mice have Decreased GABAergic Synaptic Drive

Although previous studies have demonstrated that a modest reduction in E_GABA_ can contribute to experimentally evoked seizures (Silayeva et al., 2015), reductions in GABAergic synaptic drive could also contribute to the susceptibility of Neto2-null mice to PTZ-induced seizures. To test whether Neto2^−/−^ neurons have reductions in GABAergic synaptic drive we recorded sIPSCs using the whole-cell patch clamp configuration from CA1 hippocampal neurons. We found that Neto2-null neurons had a significant reduction in sIPSC peak amplitude, compared to wild type neurons (Figures 5A,B; WT: 52.47 ± 15.97 pA, *n* = 7; *Neto2*^−/−^: 31.28 ± 6.46 pA, *n* = 8; *p* < 0.001). Neto2-null neurons also displayed a significant reduction in the frequency of sIPSCs (Figures 5A,C). In fact, only 2/8 Neto2-null neurons exhibited sIPSCs, while all wild-type neurons displayed sIPSCs; the frequency of sIPSCs is displayed in Figure 5B for neurons that displayed events.

## Discussion

Deficient GABAergic inhibition is considered to be among the chief causes for hyperexcitability underlying epilepsy (Esclapez et al., 1999; Kumar and Buckmaster, 2006). In the present study, we have demonstrated that Neto2-null mice have both a reduction in GABAergic synaptic drive and a depolarization of E_GABA_ in putative adult CA1 hippocampal pyramidal neurons compared to their wild-type counterparts. In addition, we demonstrated that Neto2-null mice (compared to wild types) have both a decrease in the latency to PTZ-induced seizures and an increase in seizure severity. Taken together we conclude that Neto2-null mice have decreased GABAergic inhibition in the hippocampus that correlates with an increased susceptibility to induced-seizures.

Mutations in KCC2 contribute towards aberrant Cl^−^ homeostasis and status epilepticus in humans and in mouse models (Hübner, 2014; Kahle et al., 2014; Puskarjov et al., 2014; Silayeva et al., 2015). Moreover, reduced KCC2 expression increases susceptibility of mice to several neurological conditions due to the decreased strength of fast hyperpolarizing synaptic inhibition (Woo et al., 2002; Tornberg et al., 2005; Kahle et al., 2008). Indeed, Tornberg et al. (2005) demonstrated that mice expressing only 15–20% of the normal KCC2 levels were more susceptible to PTZ-induced seizures. Increased propensity to PTZ-induced seizures was also reported in KCC2–1b heterozygous mice (Woo et al., 2002) expressing ~50% of normal KCC2 levels. The phosphorylation status of KCC2 at Ser940 was also recently demonstrated to critically regulate the onset and severity of status epilepticus (Silayeva et al., 2015) using a S940A mice, despite no differences in basal E_GABA_. In accordance with this finding, we observed a remarkable decrease in Ser940 phosphorylated native-KCC2 levels in neurons, and an increased severity of seizures in adult Neto2-null mice, despite a modest depolarization of basal E_GABA_. Put together, a deficit in the functional forms surface KCC2 and Ser940 phosphorylated KCC2 could contribute significantly towards a seizure phenotype in the face of epileptic challenge in the Neto2-null mice.

In addition to interacting with KCC2, Neto2 is also an auxiliary subunit of the kainate receptors (Zhang et al., 2009; Copits and Swanson, 2012; Straub and Tomita, 2012; Tang et al., 2011; Tomita and Castillo, 2012). Despite interacting with kainate receptors in the hippocampus, Neto2-null mice have intact postsynaptic kainate-mediated ionotropic synaptic transmission in pyramidal neurons (Tang et al., 2011; Wyeth et al., 2014). Therefore it is unlikely that an alteration in the postsynaptic kainate current in pyramidal neurons contributes towards the seizure phenotype we observe in the present study. However because Neto2 associates with the predominant interneuronal kainate receptor subunit GluK1 and regulates its synaptic distribution (Copits et al., 2011) it is possible that synaptic transmission from interneurons may be compromised in Neto2-null hippocampus. In accordance with this hypothesis, we observed that the frequency of spontaneous IPSCs was dramatically reduced in hippocampal neurons from Neto2-null animals when compared with WT. This indicates a reduced GABAergic drive, which could result from a reduction in presynaptic GABA release by Neto2. A reduction in presynaptic release could result from the loss of Neto2-regulation of presynaptic interneuronal GluK1 function. Additionally, the reduced sIPSC frequency could also result from decreased interneurons and/or decreased GABA release terminals in Neto2-null mice. Future studies must focus on establishing a causal link between loss of Neto2, and a loss of interneuronal GluK1 function and GABA release. The decrease in amplitude of sIPSCs could result from a decrease in postsynaptic GABA_A_R conductance, either due to changes in single channel properties or surface expression, however this possibility also awaits future examination. Thus, the combination of the disruption of postsynaptic KCC2 function and reduced GABAergic synaptic drive contributes to the PTZ-induced seizure phenotype we observed in Neto2-null animals.

We previously demonstrated that neurons in dissociated culture prepared from hippocampus of Neto2-null mice have impaired neuronal Cl^−^ homeostasis (Ivakine et al., 2013). The implications of this aberrant cellular phenotype in mature neuronal circuits remained largely untested. In the present study, we fill this important gap, by demonstrating that Cl^−^ homeostasis is also impaired in pyramidal neurons from the hippocampi of mature Neto2-null mice. Additionally, we report an overall reduced GABAergic tone in the hippocampal circuit of Neto2-null animals. Put together, the resulting dysfunctional GABAergic inhibition in these neurons could decrease the seizure threshold and increase seizure latency. Thus our study further establishes Neto2 as an important player in the maintenance of GABAergic inhibition in mature neuronal circuits.

## Conflict of Interest Statement

The authors declare that the research was conducted in the absence of any commercial or financial relationships that could be construed as a potential conflict of interest.
